# Revisiting the Marcus inverted regime: modulation strategies for photogenerated ultrafast carrier transfer from semiconducting quantum dots to metal oxides

**DOI:** 10.1039/d5ra04311e

**Published:** 2025-07-28

**Authors:** Zhexu Xi

**Affiliations:** a Inorganic Chemistry Laboratory, Department of Chemistry, University of Oxford Oxford OX1 3QR UK

## Abstract

Ultrafast charge transfer at quantum-dot/metal-oxide (QD–MO) heterojunctions governs the performance ceiling of emerging solar-energy and optoelectronic technologies. This review distills three decades of progress, covering the evolution from classical Marcus theory to modern multi-state, many-body models; the rise of exascale non-adiabatic simulations; and femtosecond spectroscopies that track electron motion in real time. Despite converging evidence for activation-less transfer under strong coupling, direct observation of the Marcus inverted regime remains scarce, largely due to Auger pathways, continuum acceptor states and interfacial defect complexity. We spotlight current strategies—outer-sphere dielectric engineering, single-charge pump–probe designs, and data–driven interface optimization—that are poised to reveal or harness inverted-region kinetics. Looking ahead, integrating low-*λ* materials, suppressing multi-carrier losses, and uniting *operando* probes with machine learning could shift QD–MO systems from kinetically limited to thermodynamically dictated performance, inspiring advances in solar fuels, infrared photodetectors and solid-state lighting.

## Abstract 2

This review bridges theory, simulations, and spectroscopy to reveal why the Marcus inverted region is elusive at quantum-dot/metal-oxide interfaces, highlighting strategies to control charge transfer for advanced solar and optoelectronic devices.

## Graphical abstract

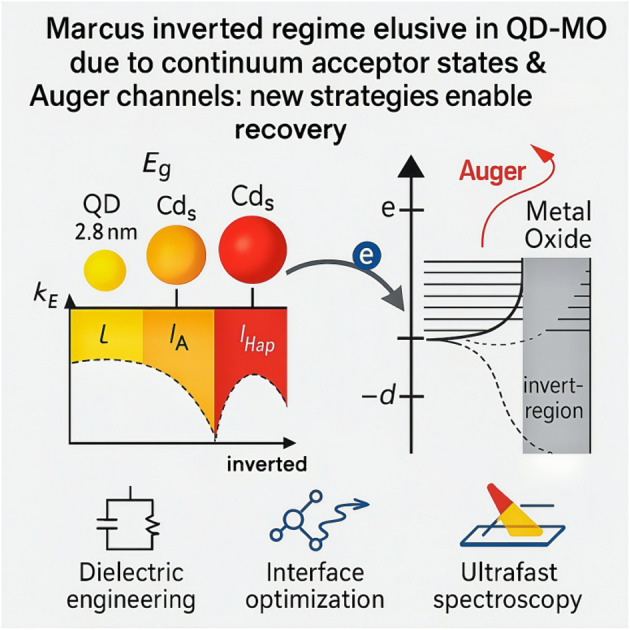

## 1 Introduction

Colloidal semiconducting quantum dots (QDs) are uniquely qualified to serve as photo-donors because their size-quantised electronic structure provides several advantages over bulk and molecular chromophores: first, their conduction-band minimum (CBM) is continuously tunable across a 1–2 eV span simply by varying their core size or composition, enabling precise control of thermodynamic driving force (−Δ*G*) for interfacial electron transfer; second, QDs exhibit exceptionally large absorption cross-sections and multiple exciton generation at high photon energies, so each particle can harvest and funnel more photonic energy than an iso-absorbing molecular dye;^1–3^ third, inorganic lattice affords their superior robustness and permits overcoating with epitaxial shells that passivate surface defects or trapping states without extinguishing the carrier wavefunctions required for tunneling.^4,5^

**Fig. 1:**
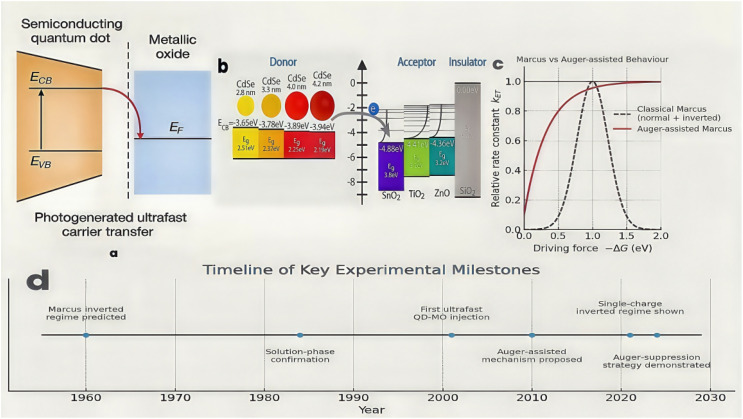
(a) Scheme of traditional electron transfer (ET) from the photoexcited QD to the MO absorbate; (b) diagram of the relative electronic energy differences between CdSe donors and MO acceptors;^6^ (c) classical *vs.* Auger-assisted Marcus ET curves (rate constant *k* – −Δ*G*); (d) illustration of timeline of experimental milestones in the discovery and subsequent definitive extension & verification of Marcus theory from 1960 to 2024. (Milestones follow the chronology established in ref. 15 and 25).

When a QD is electronically coupled to a wide-band-gap metal oxide (MO, *e.g.* TiO_2_, ZnO, SnO_2_, Nb_2_O_5_, *etc.*) matrix, a type-II heterojunction (Fig. 1a and b) is created in which photo-excited electrons can be injected from the QD conduction band into the MO continuum while the complementary holes remain in the QD for subsequent extraction.^6,7^ In addition, unlike molecular acceptors, MOs provide vast state densities and extended pathways for delocalisation, allowing injected electrons to move away from the interface on sub-picosecond timescales, thereby suppressing fast geminate recombination that would otherwise quench the light-conversion efficiency. This asymmetric separation underlies several classes of light-to-energy devices, including QD-sensitized solar cells, photoelectrochemical (PEC) water-splitting photo-anodes, and emerging hot-carrier converters.^8^

Although Marcus predicted the ‘inverted regime’ (Fig. 1c) based on the parabolic dependence of the activation barrier on (−Δ*G* + *λ*), where, once |Δ*G*| > *λ*, further exergonicity slows the reaction because the nuclear coordinates overshoot the point of intersection between the initial and final free-energy surfaces, and definitive verification (Fig. 1d for the relevant timeline of experimental milestones) has been exploited to the solid-state conjugated system, scientific hurdles abound in the fine-tuning of donor–acceptor energetics *via* QD size, composition and surface chemistry.^3^ For instance, design principles of more interactive systems, depending on engagement and aggregation of the ligand-binding domain, are elusive as a result of the electron coupling spanning six orders of magnitude; the response of MO lattice on disparate timescales determines poorly-defined outer-sphere *λ*; also, Auger-assisted electron transfer may override classical Marcus kinetics.^5,9,10^

Yet, decades of ultrafast spectroscopy on have resolved electron-injection time constants down to a picosecond or even sub-picosecond level in strongly coupled assemblies, demonstrating that the QD–MO interface can out-compete both radiative decay and Auger recombination inside the dot and accordingly contradicting Marcus' predictions.^11,12^ Size-scaling studies on CdSe and PbS dots, for instance, show that shrinking the dot (thereby increasing the driving force, −Δ*G*) monotonically accelerates the transfer, with sub-3-ps events already reported.^13^ The same interfacial physics is being exploited in photocatalysis, where QD-sensitized oxides mediate multi-electron reactions such as water oxidation or CO_2_ reduction; here, rapid extraction of the first electron is critical to build up the redox equivalents required for catalysis while minimizing back-electron transfer. In chemical sensing, QD–MO heterojunctions also provide a versatile transduction motif. Adsorption of analytes on QDs (or on the MO support) perturbs band bending and, consequently, modulates the interfacial carrier density that is monitored either resistively or capacitively. Room-temperature NO_2_ detectors based on PbS-sensitized GaAs high-electron-mobility transistors exemplify how interfacial electron transfer governs both sensitivity and dynamic range.^14^ Collectively, these application domains motivate a mechanistic understanding of carrier transfer at QD–MO junctions, especially of how thermodynamic driving force, reorganizational energy, and interfacial coupling ascertain the attainable speed and quantum efficiency.

Herein, this article re-examines ultrafast photo-induced carrier transfer dynamics from semiconducting QDs to MOs with the focus of the Marcus inverted regime, integrating theoretical constructs, first-principles/non-adiabatic simulations and ultrafast spectroscopic experiments. Specifically, we aim to:

(i) Chart the historical evolution of the Marcus theory and identify where its assumptions break down for nanoscale donor–acceptor couples;

(ii) Summarise state-of-the-art calculations that capture strong electronic coupling, multi-state acceptors and many-body excitonic effects;

(iii) critically compare experimental-rate/driving-force maps with theoretical expectations, highlighting cases where the inverted regime is suppressed or recovered;

(iv) Delineate outstanding challenges, like Auger competition, interfacial trap states, solvent and field effects, measurement time-resolution limits, and propose strategies to address them;

(v) Articulate research directions in which harnessing, rather than avoiding, inverted-region kinetics could yield superior energy-conversion efficiencies.

By providing a unified, tri-disciplinary perspective, we hope to clarify why the inverted regime is elusive in QD–MO conjugations, what its manifestation would imply for device performance, and how future experiments and models can be designed to capture the full richness of interfacial charge dynamics.

However, readers who are meeting Marcus theory for the first time may wish to scan Table 1 before proceeding; this element summarizes every variable, its physical meaning and the way it appears in the later rate expressions to provide a quick-reference scaffold that can be consulted while navigating the more advanced simulation and spectroscopy sections.

**Table 1:** One-line, plain-language definitions of key charge-transfer symbols and terms

Symbol/Term	Meaning
−Δ*G* (Driving force)	Gibbs free-energy difference between initial and final states; negative values favour forward transfer. Sets how “down-hill” the reaction is
*λ* (Reorganization energy)	Nuclear + environmental energy needed to distort from reactant to product geometry without moving the electron. Split into inner-sphere (*λ*_i_) and outer-sphere (*λ*_o_) contributions
*H*_ab_ (Electronic coupling)	The quantum-mechanical overlap between donor and acceptor states; dictating whether the system crosses the potential-energy seam adiabatically (|*H*_ab_| ≫ *k*_BT_) or tunnels incoherently (|*H*_ab_| ≪ *k*_BT_)
DOS (density of states)	Number of available acceptor energy levels per eV. A quasi-continuum DOS in oxides smears out the Marcus parabola
Exciton binding energy	Energy holding photo-generated electron and hole together inside a quantum dot; lowers the effective driving force for charge separation
Auger recombination	Non-radiative process where excess energy from one carrier annihilation excites a second carrier; competes with desired interfacial ET.
Trap state	Localised defect state that can capture and immobilise carriers, lengthening apparent lifetimes or acting as non-radiative loss channels
Dielectric confinement	Increase in coulomb interactions when a nanocrystal sits in a medium with lower permittivity, raising exciton energy and *λ*_o_
Polaron	Quasi-particle consisting of a carrier plus its self-induced lattice distortion; adds an effective outer-sphere *λ* in polar oxides
Franck–Condon window	Range of solvent configurations that conserve energy during electron transfer; its width scales with *λ* and temperature

## 2 Theoretical foundation: from the classical Marcus theory to QD–MO specific models

Electron-transfer kinetics in nanoscale heterojunctions can be understood to first order by tracking three parameters (definitions in Table 1). (i) The thermodynamic driving force, −Δ*G*, provides the energetic incentive for an electron to leave the quantum dot; increasing −Δ*G* generally accelerates transfer up to the activation-less point. (ii) The total reorganisation energy, *λ* = *λ*_i_ + *λ*_o_, quantifies how much the nuclear framework and its environment must distort to support the new charge distribution; a larger *λ* raises the free-energy barrier, slowing the reaction unless −Δ*G* is equally large. (iii) The electronic coupling |*H*_ab_| measures the quantum-mechanical overlap between donor and acceptor states; it dictates whether the system crosses the potential-energy seam adiabatically (|*H*_ab_| ≫ *k*_BT_) or tunnels incoherently (|*H*_ab_| ≪ *k*_BT_). The interplay of these three levers—energetic bias (−Δ*G*), nuclear flexibility (*λ*) and wavefunction communication (|*H*_ab_|)—governs whether a given interface operates in the normal, activation-less or inverted regime and therefore underpins every experimental trend and simulation result discussed in the rest of this review.

### 2.1 Classical description of reorganizational energy (*λ*), driving force (−Δ*G*) and electronic coupling (*H*)

**Fig. 2:**
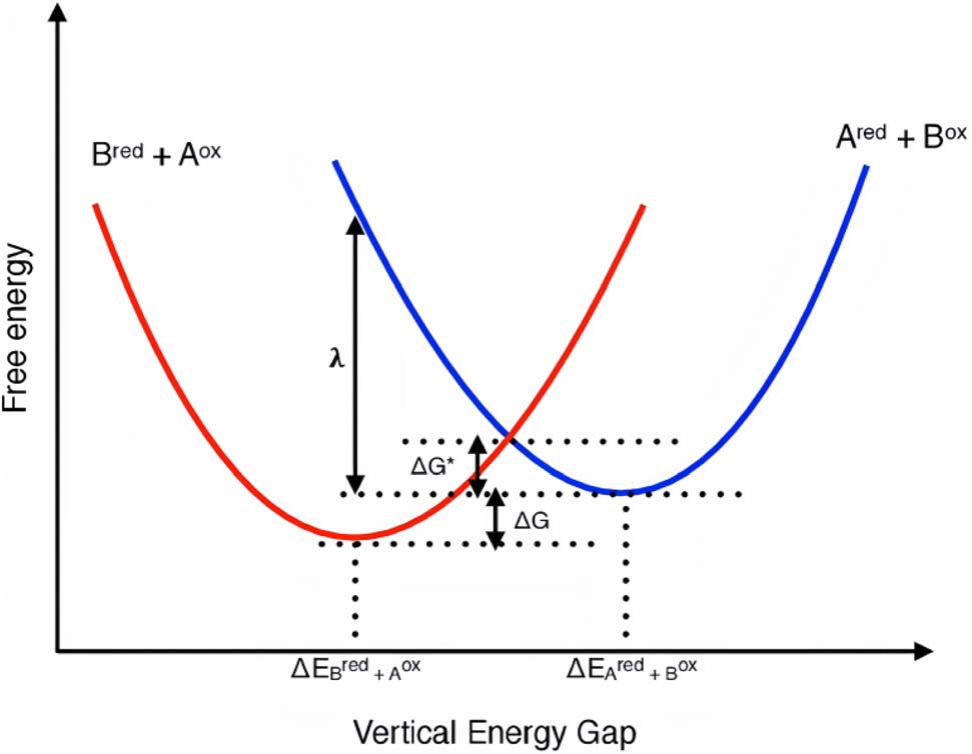
Illustrative Marcus plot showing the two parabolic free energy curves (representing normal and inverted driving forces) as a function of ΔE for the electron transfer between two redox compounds A and B.^18^

Electron transfer (ET) or hole transfer (HT, or back-electron transfer) in condensed matter is most conveniently visualized on a two-dimensional free-energy surface spanned by a collective “solvent” (or nuclear) reaction coordinate, *Q*. Within the harmonic approximation invoked by Marcus, the potential of the reactant and product states is represented by two parabolas of identical curvature (Fig. 2) that intersect at a certain nuclear configuration.^15^ The vertical separation of the minima of those parabolas is the standard Gibbs free-energy change of the reaction, Δ*G*°, usually expressed as the ‘driving force’ Δ*G* = −Δ*G*° when discussing photoinduced processes.^15,16^ The reorganizational energy *λ* is defined as the energetic penalty required to distort the nuclear configuration of the donor–acceptor pair (and its environment) from its initial equilibrium geometry to the equilibrium geometry of the final state without altering the electronic population. Operationally, *λ* is the sum of an inner-sphere component (bond-length, bond-angle adjustments within the reacting species) and an outer-sphere component (polarization of the surrounding lattice, solvent shells, or surface dipoles).^17^ For a better understanding, numerical ranges of key ET-relevant parameters of several typical material classes have been presented in Table 2.

**Table 2:** Typical parameter ranges in representative QD–MO systems

Material class	−Δ*G* (eV)	*λ*_i_ (eV)	*λ*_o_ (eV)	*λ*_total_ (eV)	|*H*_ab_| (meV)	*τ*_ET_ range
CdSe QDs/TiO_2_ (II–VI)	0 → −0.7	0.05 – 0.07	0.15 – 0.25	0.20 – 0.30	1 – 10	0.5 – 5 ps
PbS QDs/TiO_2_ (IV–VI)	0 → −0.5	0.04 – 0.06	0.10 – 0.20	0.14 – 0.24	2 – 15	0.2 – 2 ps
CsPbBr_3_ QDs/SnO_2_ (perovskite)	0 → −0.4	0.03 – 0.05	0.08 – 0.15	0.11 – 0.20	5 – 25	0.1 – 1 ps
Gr-QD hybrids	0 → −0.3	≤0.03	0.05 – 0.07	0.08 – 0.10	10 – 40	<200 fs

In the weak-coupling or non-adiabatic limit—the regime most thoroughly treated by classical Marcus theory—the electronic coupling matrix element *H*_ab_ between donor (a) and acceptor (b) is small relative to vibronic energy spacings. Application of Fermi's Golden Rule leads to the celebrated rate expression,1
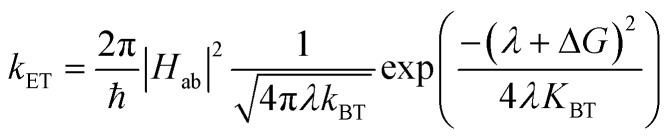


Which captures, in closed analytic form, how structure (through *H*_ab_) and thermodynamics (through *λ* and Δ*G*) conspire to determine CT kinetics.

Two distinct kinetic regimes emerge from this expression.^19,20^ In the normal region (−Δ*G* < *λ*) the activation barrier decreases monotonically as the driving force grows, so *k*_ET_ increases almost exponentially. When the driving force exceeds the reorganizational energy (−Δ*G* > *λ*) the exponential term dominates with a positive exponent, causing the rate to decrease again; this counter-intuitive behaviour defines the Marcus inverted region. The barrierless maximum at −Δ*G* ≈ *λ* marks the transition between the two regimes and, in principle, sets an upper limit on how fast a single-step, non-adiabatic electron transfer can proceed at a given temperature.

The prefactor ∣*H*_ab_∣^2^ embeds molecular-scale details of the donor–acceptor electronic overlap and falls off approximately exponentially with distance in through-space tunneling or as a function of bridge length in through-bond super-exchange. When ∣*H*_ab_∣ becomes comparable to or larger than the vibrational bandwidth, the assumption of weak coupling breaks down: the system approaches the adiabatic limit, the nuclear coordinate follows a single, smoothly connected potential-energy surface, and the reaction rate is no longer given by the Golden-Rule expression but by the frequency with which the system crosses the nuclear barrier (often on the order of phonon frequencies).^21,22^

These classical constructs, *λ*, Δ*G* and |*H*_ab_|, provide the indispensable language for analyzing carrier transfer across any redox interface. In QD–MO heterojunctions, however, each term acquires new physical nuance: (i) *λ* may be dominated by outer-sphere polarization of an extended oxide lattice rather than solvent shells; (ii) Δ*G* couples discrete quantum-confined states with a quasi-continuous metal-oxide conduction band, complicating its definition; and (iii) |*H*_ab_| can traverse from non-adiabatic to strongly adiabatic due to direct anion–cation orbital overlap at epitaxial interfaces.^23^ The remainder of this review will show how these deviations from molecular paradigms condition the observability of the Marcus inverted region, as well as how new theoretical extensions, simulations and ultrafast experiments strive to capture them quantitatively.

### 2.2 QD–MO interface: multi-state and strong coupling characteristics

This section pinpoints how the classical single-state Marcus picture must be generalized when a zero-dimensional semiconductor nanocrystal injects an electron (or a hole) into the quasi-continuum of states that form the conduction (or valence) band of a crystalline metal oxide. We focus on three inter-related facets: (i) the continuum of acceptor states, (ii) the way quantum-confinement tunes driving force Δ*G*, coupling |*H*_ab_| and reorganizational energy *λ*, and (iii) the redistribution of inner-(*λ*_i_)and outer-sphere contributions (*λ*_o_) to *λ* (defined as *λ*_i_ + *λ*_o_) that accompanies rigid inorganic donors and polarizable oxide acceptors. We close by discussing how strong donor–acceptor coupling pushes QD–MO systems to the edge of the non-adiabatic/adiabatic divide, with direct consequences for the observability (or absence) of the Marcus inverted regime.

#### 2.2.1 Quasi-continuum of acceptor states

**Fig. 3:**
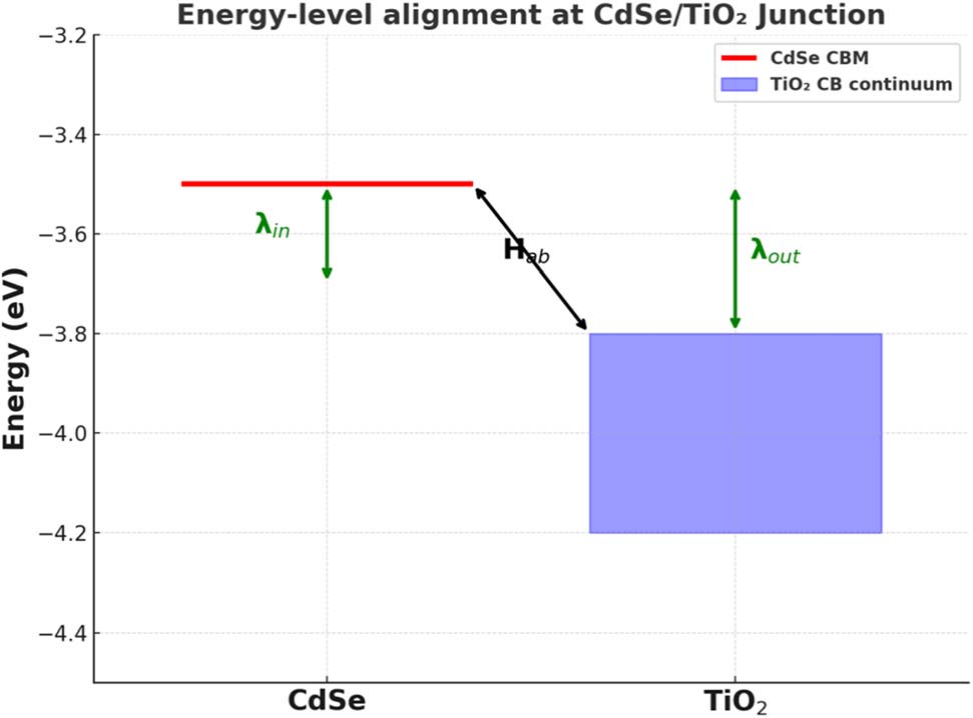
Schematic of energy-level alignment at a CdSe/TiO_2_ junction showing donor CBM, oxide continuum, reorganization energy components *λ*_in_ and *λ*_out_, and electronic coupling *H*_ab_.

Unlike molecular systems, where the density of states (DOS) of molecules (typically orbitals of a single molecule or a finite set of molecular orbitals) are discrete, the conduction band of metal oxides provides a quasi-continuous spectrum of available states. This is particularly relevant at the nanoscale, where the MO's conduction band becomes more finely structured, and the DOS at specific energy levels can strongly influence the electronic transfer dynamics, where the continuous nature of the MO's conduction band allows for a broader range of energy states that the photogenerated electron can occupy once injected from the QD.^4,24^ This feature increases the overlap between the electronic states of QD and conduction band of MO, accordingly reducing the energy mismatch (at a meV level, far smaller than *k*_B_*T*) and leading to a more efficient ET process. Furthermore, these states can be further modulated by engineered oxide materials through doping, surface functionalization, or nanostructuring, thereby yielding a more complex, multi-state Marcus model:2
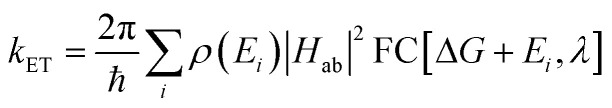
Here the FC term represents the Franck–Condon factor, retaining the Gaussian Marcus shape centred at Δ*G* = −*λ*, but the total effect with *ρ*(*E*_i_) smears and skews the parabola. Early transient-absorption studies by Scholes and coworkers on CdSe@TiO_2_ explicitly reproduced the sharp rise in *k*_ET_ at small |Δ*G*| followed by only a weak saturation instead of the expected downturn, matching the theoretical prediction for a discrete-to-continuum problem.^6,25^Fig. 3 below clarifies how confinement and dielectric environment reshape the Marcus parameters in this settings. Density-functional tight-binding (DFTB-MD) simulations subsequently showed that the effective *λ* extracted from such integrals is smaller (≈100–150 meV) than the ≈0.6 eV typical of molecular donors because the oxide continuum offers a resonant state almost irrespective of instantaneous solvent polarization.^26^

A crucial corollary is that the Marcus inverted peak is flattened as the continuum integral ‘channels’ excess driving force into progressively higher-lying oxide states instead of raising the activation barrier.^7^ This continuum-averaging mechanism is separate from but additive to the Auger-assisted bypass discussed in Section 5; this mechanism operates even for single-charge transfer events and therefore has to be built into any realistic kinetic model.

#### 2.2.2 Quantum-confinement tunability

The bandgap modulation of a QD, which governs the energy required to excite an electron from the valence band to the conduction band, is strongly dependent on its size. The first exciton energy of a II–VI nanocrystal scales roughly as *E*_g_(*R*) ∝ *R*^−2^. Due to the quantum confinement effect, the QD conduction-band edge is blue-shifted by hundreds of meV as the particle radius contracts, therefore facilitating the ET rate with optimized −Δ*G* for electron injection. Reducing the diameters of II–VI QDs (CdSe, CdS, CdTe) from 5–6 nm to 2–3 nm raises their conduction-band edge by ≈0.3 − 0.6 eV, providing a larger thermodynamic push for injection into TiO_2_ or ZnO.^27,28^ CdTe dots linked to TiO_2_ films show a three-to-five-fold rate increase and higher photocurrent at 2.5 nm *versus* 4 nm, consistent with their larger band-edge offset.^29^ This tunability has been the workhorse for experimental ‘driving-force series’, but because the accessible window often stops short of −Δ*G* ≈ *λ*, most size-series fail to enter the inverted regime.

Besides, tuning the QD size can optimize the alignment between the QD excited state and the MO conduction band; in turn, this tuning effect can enhance the CT efficiency. With reduced QD core sizes, the corresponding blue-shift shrinks the real-space envelop of the electron wavefunction, subsequently making the donor orbital acquires non-negligible amplitude on surface atoms that are chemically bonded to the oxide and thereby inducing a larger exciton-phonon coupling strength for an enhanced strong-coupling/adiabatic system.^7,30^ The twin effects of larger −Δ*G* and *H*_ab_ are to shorten the injection time from hundreds of femtoseconds in large dots to <10 fs in strongly confined dots; PbS@TiO_2_ heterojunctions constitute the current speed record with *τ*_ET_ = 6.4 ± 0.4 fs, a hallmark of adiabatic, barrier-free transfer.^31^

The strong-coupling limit is therefore frequently reached at QD–MO interfaces, where the delocalized QD electron overlaps directly with itinerant oxide states or couples through only an angström-scale ligand. Landau–Zener analysis shows that when *H*_ab_ exceeds ∼50 meV the activation barrier collapses, and the system crosses to the adiabatic branch of the Marcus parabola; under these conditions nuclear motion no longer gates the transfer but merely modulates a rate that is already near the vibrational timescale.^32^ This regime is largely responsible for the oft-noted absence of an inverted region in excitonic QD injections, a point revisited in Section 5.

Size effects are not limited to the QD alone but also impact the MO material. Nanostructuring the oxide, such as by using nanoparticles or nanosheets, can increase the surface-to-volume ratio and, consequently, the number of active sites available for electron transfer.^6,33^ This enhancement in surface area allows for better alignment and overlap between the quantum dot and the oxide's conduction band, facilitating faster charge transfer rates. Thus, the combination of size-dependent effects in both the QD and the metal oxide becomes an essential consideration when designing efficient QD–MO systems for applications such as photovoltaics, photocatalysis, and energy storage.^34^

#### 2.2.3 Redistribution of reorganizational energy: inner- and outer-sphere contributions (*λ*_i_*vs. λ*_o_)

In traditional Marcus theory, *λ* is the energy required to reorient the solvent and the nuclei during the electron transfer process. *λ* is typically divided into two components: the inner-sphere component *λ*_i_, which is associated with the donor molecule's intrinsic vibration, and the outer-sphere component *λ*_o_, which corresponds to the reorganization of MO's environment like its lattice and solvent interactions.

In QD–MO conjugations, *λ* can be hugely influenced by the core size and structure of QD as well as the nature of MO surface. In systems where QD is deeply anchored to the MO surface, the electronic reorganization associated with the QD's excited state is minimized due to the fixed position of the QD. Colloidal QDs possess rigid, inorganic lattices in which bond-length relaxation or distortion upon surface redox reaction is minimal; reported *λ*_i_ values are ≤70 meV, an order of magnitude lower than those of molecular dyes.^7,35^ Conversely, the *λ*_o_ term can dominate due to surface ligands, solvent polarity and the polaronic dielectric response of the oxide lattice. In principle, a nanocrystal embedded in a lower–permittivity medium experiences an image-charge self-energy that raises its exciton energy and at the same time reduces its charge-screening ability, marginally increasing *λ*_o_.^36^ Yet when the dot directly contacts a high-κ oxide (TiO_2_, *ε* ≈ 60), the self-energy drop can overshoot the dielectric confinement penalty, yielding an overall lower *λ* than in aprotic solvents. Another first-principles calculations on graphene-QD@azobenzene and related hybrid interfaces place *λ*_total_ in the range of 0.08–0.10 eV, with only ∼50 meV originating from internal nuclear relaxation,^37^ where this redistribution means that engineering the ligand shell or embedding medium can tune *λ* more effectively than altering the QD core itself.

In addition to dielectric confinement, MO's polar phonons could be another potential parameter for modulating the outer-sphere term. Conduction-band electrons in anatase TiO_2_ couple strongly to longitudinal optical phonons (ℏ*ω* ≈ 44 meV). Polaron formation during or immediately after injection therefore adds an effective *λ*_o_ to the FC window. Recent femtosecond THz studies demonstrate that the 100 fs rise of the free-carrier signal in PbSe@TiO_2_ is limited by polaron dressing rather than by the tunnelling step itself.^26,38^

Combining these contributions, the composite *λ* for QD–MO injection often falls into the 150–250 meV range, which are roughly half the value typical for dye-sensitized analogues, and therefore moves the maximum of the Marcus parabola to larer −Δ*G*. In practice, this means a 2 nm CdSe QD would need to supply ≈0.25 eV extra driving force over the 5 nm counterpart to reach the same *λ*-limited optimum. Absence of such additional headroom is one reason why many size-series never probe the inverted side.

#### 2.2.4 Effect of Δ*G* on ET beyond size-tuning

As discussed above, the drawback of the effect of Δ*G* on interfacial ET as a function of the core size of QD is the lack of fitted data not viable to a wide range of energies so that the correlation between Δ*G* and ET cannot be fully interpreted and applied to the multi-state Marcus theory.^39^ Consequently, more revelation on the effect of bandgap modulation on ET should be highlighted without tuning the core size of QD or the electronic nature of MO.^6,39,40^ In the multi-state Marcus framework, the core of optimizing Δ*G* is lowering the activation barrier, directly linked with the overlap of electronic state of QD and the conduction band of MO, provided that the total *λ* and *H*_ab_ remain comparable. In QD–MO heterojunctions, the continuum of MO states flattens the top of the Marcus parabola, but the normal-to-inverted crossover is still detectable when Δ*G* is tuned over ≳0.5 eV.^7^

The first approach is surface-ligand engineering, where introducing X-type or π-conjugated organic ligands can provide interfacial dipoles that rigidly translate QD band edges. Solution-phase exchange of the native oleate shell for ligands with large permanent dipoles (*e.g.* fluorinated cinnamates) can shift the CBM of PbS by >2 eV without altering QD size.^41^ The induced surface dipole lowers (or raises) the donor level relative to the MO's CBM, linearly scaling −Δ*G* at ≈ 0.3 − 0.5 eV·D^−1^.^42^ In time-resolved THz measurements this chemical gating accelerates *k*_ET_ by an order of magnitude, until saturation is reached when the FC window starts to overlap densely with oxide states. Meanwhile, here negative dipoles can reduce −Δ*G* to probe the weak-driving side.

Altering the oxide environment like solvation could change the self-energy of carriers in nanocrystals by specific solvent-surface ion interactions and dielectric screening, consequently enabling *in situ* tuning of Δ*G* without chemical modification; also, an dielectric environment is useful for ‘on-solvent’ kinetic titrations that map the Marcus parabola under otherwise identical interfacial chemistry. Spectroelectro-chemistry shows ≈1 eV band-edge shifts between polar and non-polar solvents.^43^

Electro-injection of carriers from electrolyte or ionic-liquid gating shifts the Fermi level of QD by over 1 eV; the same junction probed under bias therefore experiences a continuous, *operando* change of Δ*G*.^44,45^ Naturally, this variation can transiently drive the system into the inverted regime before structural relaxation sets in. For single QDs addressed in transistor geometry, an external field of only 0.3 MV cm^−1^ changed *k*_ET_ by a factor of ∼4000, in agreement with field-induced band bending predicted by dissipative tunneling theory.^46^

Employing bridging molecules in donor–bridge–acceptor enegetics could also regulate the rate constant without altering the QD size. One typical example is replacing long alkanedithiol linkers with *n*-phenylene bridges that deepens the acceptor LUMO and simultaneously enhances electronic coupling. The relevant Pump-THz spectra shows that shortening the bridge from *n* = 11 to *n* = 4 augments −Δ*G* by ∼150 meV and speeds up *k*_ET_ by over one order of magnitude at constant dot size.^47^

An additional example of Δ*G* modulation without tuning the QD size is treating Δ*G* as a function of pH that the whole system was immersed.^48^ Increasing pH could lower the oxide conduction-band edge to a more negative potential, consequently decelerating Δ*G* and the CT rate. Besides, protonation of oxide surface hydroxyls lowers the oxide CB edge, whereas deprotonation raises it; varying the pH between 3 and 11 tunes Δ*G* by ≈0.25 eV, with *k*_ET_ responding exponentially until the Marcus peak is approached.^49,50^ pH-controlled assembly additionally dictates QD loading density, indirectly influencing Δ*G via* inter-dot dipolar fields.

Very large −Δ*G* values (<−0.6 eV) favour Auger-assisted transfer, comprising steps like transient photo-charging and exciton multiplicity, over Marcus tunneling, maintaining a monotonic rise of *k*_ET_ even beyond the classical peak; this effect is amplified in photocharged or multiexcitonic dots. Under high-flux illumination QDs accumulate photoholes, raising the quasi-Fermi level of the electron and enlarging −Δ*G* for the second (multiexciton) electron to eject. THz conductivity studies on CdSe@ZnO show that biexciton injection is 3× faster than single-exciton injection, a trend reproduced by size-dependent Marcus fits that incorporate the extra driving force and Auger energy redistribution.^7,43^

## 2.2.5 Summary

In summary, the coexistence of a dense acceptor continuum, large and tunable *H*_ab_, and a small but environment-sensitive *λ* produces rich energetic landscapes. When −Δ*G* < *λ*, the rate is *λ*-controlled and highly sensitive to solvent or surface phonons; when −Δ*G* ≫ *λ*, the system can either enter the continuum-limited plateau predicted by the many-state model or bypass the inverted region altogether *via* Auger-assisted channels that redistribute excess energy within the exciton manifold. Recognizing which regime applies to a given QD–MO pair is essential for rational interface design and for interpreting ultrafast spectroscopic data. Recognizing and quantifying each contribution is indispensable for any attempt to ‘revisit’ Marcus theory under the ultrafast conditions that prevail at semiconductor heterointerfaces in modern energy-conversion schemes.

### 2.3 Auger-assisted mechanisms and the disappearance of the Marcus inverted region

#### 2.3.1 Auger-assisted charge transfer mechanism

**Fig. 4:**
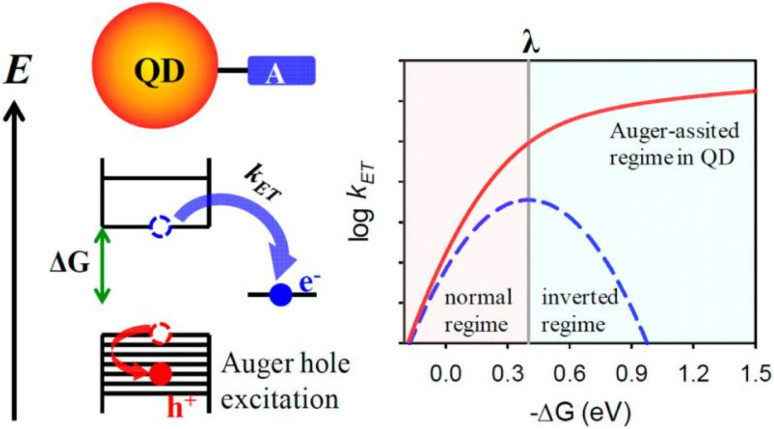
Illustration of Auger-assisted charge transfer in QD–acceptor systems. Left panel: photo-excited electron (blue) transfers to the acceptor with rate constant *k*_ET_, using excess energy to excite a hole (red) *via* Auger processes while the right panel: comparison between classical Marcus theory (blue dashed) showing a clear inverted region, and the Auger-assisted scenario (red solid), where the inverted region effectively disappears, enhancing ET rates at high driving forces.^25^

Experimental surveys of electron injection from colloidal QDs into MO acceptors have long reported a monotonic acceleration of the transfer rate with increasing −Δ*G*, even when −Δ*G* clearly exceeds typical reorganizational energies *λ* predicted for these interfaces.^6,48^ Such behaviour contradicts classical Marcus theory, which requires the rate to decline once −Δ*G* > *λ*, *i.e.* to enter the inverted regime (Fig. 4). The absence of this turnover prompted a re-examination of the elementary energetics operative in quantum-confined donors, indicative of alternative mechanisms at play.^6,32,49^

In QDs, strong confinement and enhanced coulomb interactions markedly facilitate Auger recombination processes, wherein excess electronic energy is non-radiatively transferred to another electron or hole to a higher valence-bond level instead of being dissipated vibrationally as heat or radiative recombination.^25,32^ Consequently, this Auger-type energy-exchange opens a continuum of product states with effective driving forces ΔG* = Δ*G* + *E*_h_, where *E*_h_ is the hole-excitation energy.^48^ For each Δ*G* there therefore exists at least one channel in which |ΔG*| ≈ *λ*, maintaining barrier-less transfer and erasing the inverted branch predicted for a single-electron coordinate. Formally, the rate expression becomes a convolution of the standard Marcus kernel with the density of accessible hole states *ρ*(*E*_h_) and a three-body electronic coupling term *H*_AET_(*E*_h_) (AET means Auger-assisted ET).^50,51^

#### 2.3.2 Simulation evidence

Time-domain non-adiabatic molecular dynamics and *ab initio* many-body perturbation theory show that Auger-mediated channels dominate whenever the electron–hole coulomb energy *J*_e–h_ exceeds the electron–phonon coupling.^51^ Direct simulations on CdSe and PbS QD–MO clusters reveal femtosecond ET accompanied by hole promotion, reproducing the plateau-like rate *vs.* Δ*G* observed experimentally without invoking unphysically small *λ*.^22^ These calculations further predict a characteristic scaling *k*_AET_ ∝∣*H*_AET_∣^2^*ρ*(*E*_h_), linking the strength of the coulomb exchange and the density of intraband hole states to the persistence of the normal-like regime.^51^ Notably, the Auger channel is suppressed once the donor carries a single charge instead of an exciton, providing a route to ‘switch off’ the bypass.^49,52^

#### 2.3.3 Experimental evidence

Systematic transient-absorption studies spanning >1 eV in driving force from CdS/CdSe/CdTe QDs to several bridging molecules like anthraquinone, methyl-viologen and methylene-blue show that *k*_ET_ rises four orders of magnitude without turnover, matching quantitatively an Auger-assisted rate model with *λ* ≈ 0.4–0.5 eV.^25,48^ Ultrafast THz spectroscopy on PbSe@SnO_2_ and CdSe@TiO_2_ assemblies confirms that transfer remains sub-100 fs even at −Δ*G* ≈ 1.2 eV, again inconsistent with classical inversion.^6,53^ Conversely, when Wu *et al.*^31^ engineered single-charge donor states by photo-populating adsorbed-molecule-QD complexes, the long-sought inverted regime re-emerged at −Δ*G* ≈ *λ*, validating that the excitonic Auger pathway, not an exotic *λ*, accounts for its previous invisibility. Complementary single-particle fluorescence intermittency measurements further reveal that QDs exhibiting longer ‘bright’ durations, indicative of rapid hole extraction and thus weaker electron–hole coupling, begin to display incipient inverted behaviour, tightly correlating Auger strength with Marcus deviation.^48^

#### 2.3.4 Implications for modified Marcus theory at QD–MO interfaces

Taken together, these findings require extending Marcus theory into a multi-exciton framework where electronic coupling is a tensor linking donor, acceptor and spectator charges. The effective reorganizational coordination becomes multidimensional, mixing nuclear and coulombic degrees of freedom, while the rate surface collapses to the classical parabolic form only when the electron–hole coulomb energy gets closer to zero.^5,51^ Practically, the analysis underscores that suppressing Auger exchange, *via* selective hole scavengers, type-II band alignment, or dielectric screening, will not only reveal the authentic Δ*G*-dependence but may also enable deliberate placement of back-transfer into the inverted regime to prolong charge separation.^54^ Moreover, the Auger concept rationalizes why hot-electron cooling in QDs is similarly accelerated: excess kinetic energy is off-loaded to the hole, circumventing the phonon bottleneck, another manifestation of an inverted-like barrier solved by coulomb exchange.^32^ Overall, Auger-assisted transfer provides a coherent, experimentally validated explanation for the vanishing inverted regime at QD–MO interfaces and establishes new handles for kinetic control in nanostructured energy-conversion systems.

## 3 Advances in computational simulations

### 3.1 DFT/TD–DFT predictions of band alignment and electronic coupling at QD–MO interfaces

Density-functional theory (DFT) and time-dependent DFT (TD–DFT) have become indispensable for mapping the energetic landscape and electronic coupling that govern photo-induced carrier transfer across QD–MO interfaces. Early plane-wave calculations on PbSe@TiO_2_ prototypes showed that quantum confinement and surface dipoles shift the PbSe conduction edge 0.3–0.5 eV above the TiO_2_ CBM, thus establishing a downhill driving force for electron injection.^55,56^*Ab initio* non-adiabatic molecular dynamics (NAMD) subsequently revealed that this offset, combined with donor–acceptor delocalization, supports sub-100 fs adiabatic injection dominated by optical phonons rather than thermally activated Marcus hopping.^51^

Facet engineering on the oxide side provides further modulation. Azpiroz *et al.*^56^ compared rutile (001) and (101) slabs and found that (101) confers nearly twice the electronic coupling matrix element, shortening the calculated injection time to ∼1 ps, albeit at the cost of faster back-electron transfer. Parallel TD–DFT DOS analyzes link the stronger coupling on (101) to the emergence of surface-projected Ti 3d states that resonate with the PbSe donor orbital manifold, a feature absent on (001) facets.^5,57^ Similar geometric sensitivity appears in graphene-QD@TiO_2_ (011) heterostructures, where DFT + U predicts covalent C–O–Ti bridges that widen the interfacial bandwidth and red-shift the absorption edge, boosting visible–light activity.^58^

Surface chemistry exerts a complementary handle on band alignment. Ligand-exchange calculations demonstrate that substituting long-chain carboxylates with halide or short-inorganic ligands can swing the PbSe Fermi level by >0.4 eV and even flip the offset from type-I to type-II, thereby altering the preferred carrier-transfer direction.^58^ Such dipole-driven shifts rationalize experimental reports of accelerated electron extraction after thiocyanate or chloride treatment in QD solar cells. A recently proposed wave-function-projection scheme provides a transferable route to determine absolute band edges for large QD–MO supercells, avoiding spurious vacuum-level alignment and reproducing experimental offsets within 0.1 eV.^59^

Beyond Pb-chalcogenides, CdSe@SnO_2_ has emerged as a test-bed for electron transfer layers of MO in perovskite and QD devices. Hybrid-functional calculations place the CdSe conduction edge only 0.15 eV above SnO_2_, implying a smaller driving force but reduced lattice mismatch compared with TiO_2_.^60^ Stress-dependent DFT studies^61^ further predict that tensile strain at the SnO_2_ surface lowers its CBM, completely eliminating the offset under 2% biaxial tension and thus explaining the experimentally observed sensitivity of injection efficiency to annealing-induced stress. Defect calculations indicate that interfacial Sn vacancies generate shallow donor states that act as ‘stepping-stones’ for electrons, enhancing coupling yet risking recombination if unpassivated.^61^

Methodologically, the field is shifting toward machine-learning-assisted excited-state dynamics. Interpolated NAMD Hamiltonians trained on sparse TD–DFT snapshots can now propagate ∼10 ps trajectories for ∼10 nm heterostructures at hybrid-functional accuracy, capturing polaronic relaxation and hot-electron transfer in PbSe@TiO_2_ within realistic device geometries.^62^ Coupled with real-time TD–DFT, these approaches predict that hot injection can out-compete Auger cooling when the interstate coupling exceeds 50 meV, offering a quantitative design rule for exploiting excess photon energy.

DFT and TD–DFT studies have progressed from static band diagrams to chemically specific, time-resolved pictures of carrier transfer, essential for the rational design of QD–MO interfaces in optoelectronic devices. By tailoring QD sizes discussed above, facet selection, surface ligand engineering, and stress tuning, it is possible to optimize band alignments and electronic couplings to achieve desired CT characteristics, fundamentally suppressing Marcus barriers and driving the system into an adiabatic or near-ballistic regime. Ongoing developments in scalable excited-state dynamics promise to generalize these insights to more complex mixed-dimensional and multi-particle interfaces.

### 3.2 Non-adiabatic molecular dynamics (NAMD) and real-time TD–DFT

First-principles non-adiabatic simulations have become indispensable for visualising how photoexcited carriers leave QDs and enter metal–oxide acceptors on the 10–100 fs time-scale. *Ab initio* NAMD shows that strong donor–acceptor coupling and specific interfacial phonons can overcome electron–phonon dissipation and realise Marcus-type transfer without an activation barrier, while emerging machine-learning and exascale real-time TD–DFT (RT–TDDFT) frameworks now push system sizes toward experimentally relevant heterostructures. These advances reveal why the Marcus inverted regime remains elusive under ordinary excitonic conditions and how vibrational mode engineering or Auger management can tip the balance.

The earliest *ab initio* NAMD work on PbSe@TiO_2_ established a benchmark electron-injection time of ≈30 fs, fast enough to out-race intradot cooling and to proceed adiabatically owing to a ≳100 meV interstate coupling.^50^ Subsequent studies on CdSe, ZnSe and hybrid perovskite nanocrystals confirmed that QD–MO contacts routinely operate in the strong-coupling limit, shifting the reaction coordinate from solvent-dominated outer-sphere reorganization to lattice-driven inner-sphere dynamics.^7,63^ These simulations reproduce the experimentally observed size-dependent acceleration of electron transfer and simultaneously rationalize why the anticipated Marcus turnover is masked when the hole remains on the dot.

Interfacial vibrational modes emerge as critical gatekeepers. High-frequency polar optical phonons in both the QD and TiO_2_ rapidly modulate the donor–acceptor diabatic gap, creating ‘targeted resonances’ that channel the wave-packet across the interface within a vibrational period.^51^ In contrast, low-frequency acoustic and surface modes mainly broaden the distribution of initial conditions, accounting for single-particle rate heterogeneity.^64^ Nuclear quantum effects (NQEs) included through path-integral surface hopping further accelerate the separation by enhancing tunneling along curvilinear coordinates, suggesting that isotopic substitution or phonon-mode confinement could become design knobs for approaching the inverted regime experimentally.^64,65^

Methodological innovations now extend NAMD beyond a few hundred atoms. Linear-scaling RT–TDDFT with classical nuclei allows Ehrenfest or surface-hopping propagation of QD–bridge–MO supercells for several picoseconds, explicitly tracking charge density flow and vibrational energy redistribution.^66^ Machine-learning potential surfaces trained on high-level data reduce the electronic-structure overhead by two orders of magnitude, enabling statistically converged trajectories for multi-layered composites and alloyed QDs.^67^ The open-source QRCODE (Quantum Research for Calculating Optically Driven Excitations) engine released in 2024 provides exascale RT–TDDFT throughput and automated analysis of time-resolved charge populations, making systematic screening of ligand chemistry or oxide polymorphs practical.^68^

Crucially, multi-electron extensions of NAMD illuminate the interplay between Auger recombination and interfacial transfer. Simulations that allow two electrons and one hole show that intradot Auger scattering can furnish the excess energy required for injection even when −Δ*G* > *λ*, thereby flattening the Marcus parabola and delaying the inverted regime turnover.^25^ Hot-electron trajectories in PbSe QDs molecularly bridged to TiO_2_ predict a 6 fs injection before thermalization, but only when the bridge provides >50 meV electronic coupling and the phonon bath is weakly coupled.^69,70^ These findings emphasize that separating charges on femtosecond scales is necessary, but not sufficient, to observe the inverted regime; the system must also inhibit multi-carrier energy shunts.

Despite the progress, challenges remain. None of the current schemes fully incorporate long-range oxide polarons, defect states or stochastic environmental fluctuations that can slow injection in real devices.^71,72^ Integrating explicit dielectric embedding with RT–TDDFT or coupling NAMD to kinetic Monte–Carlo frameworks for microsecond evolution will be essential to bridge the gap to experiment. Nevertheless, the convergence of accurate non-adiabatic theory, scalable algorithms and high-resolution spectroscopy now offers an unprecedented opportunity to map design parameters (*e.g.* coupling, vibrational spectrum, and many-body interactions) onto Marcus free-energy landscapes and to engineer QD–MO junctions that deliberately operate at, or even exploit, the inverted regime.

### 3.3 Multiexciton and hot-phonon models: emerging many-body perspectives

Recent simulations have moved beyond single-particle Marcus pictures and now treat many-body carrier multiplication, hot-phonon-limited cooling and non-Markovian open-system dynamics on equal footing.^73^ State-of-the-art *ab initio* NAMD confirm that strong electronic coupling at QD–MO interfaces can outrun lattice thermalization, while quantum-master-equation (QME) formalisms reveal how memory effects shape the Marcus inverted regime.^5,51^ Crucially, models that include multiple-exciton generation (MEG), hot-electron transfer (HET) and polaronic phonon dressing collectively handle several experimental puzzles: (i) the apparent absence of the inverted regime under high pump fluence, (ii) exceptionally long (100 ns) hot-electron lifetimes recently reported under electrical bias,^74^ and (iii) size-dependent branching between Auger recombination and ultrafast injection.

#### 3.3.1 Multiple-exciton generation and dissociative transfer

Early MEG theories treated intradot Auger decay as a loss channel, but modern GW–Bethe–Salpeter equation (GW–BSE) + NAMD simulations show that Auger interactions can mediate electron injection by partitioning excess energy between carriers and the oxide manifold.^7,75^ Prezhdo and co-workers demonstrated that a bi-exciton created in a 3 nm PbSe QD injects its first electron into TiO_2_ within 40 fs, leaving the second exciton cold enough to survive long enough for subsequent transfer.^75^ Real-time Ehrenfest dynamics extended to >1 ps suggest that the MEG yield required to surpass the Shockley–Queisser limit (*η*_MEG_ ≈ 1.2) is attainable only when the reduced reorganization energy *λ* < 100 meV, a regime accessible in rigid, halide-passivated QDs.^76,77^ Experimentally, nano letters^78^ reported 112% electron harvesting efficiency for MEG in PbS QDs anchored on TiO_2_, validating the model predictions. These studies converge on a key design rule: driving the first step of MEG-assisted transfer slightly past Δ*G* ≈ *λ* maximizes the branching ratio towards injection while keeping the back-transfer in the Marcus inverted zone.

#### 3.3.2 Hot-electron and hot-phonon bottleneck effects

HET provides an alternative route to beat thermal losses. *Ab initio* NAMD on PbSe@TiO_2_ predicts a 25 fs injection of carriers situated >0.4 eV above the TiO_2_ CBM, preceding carrier-phonon equilibration.^75,79^ Parallel transient-absorption experiments recorded unit-yield HET from strongly coupled PbS QDs at room temperature, implying an interfacial coupling |*H*| > 100 meV.^80^ Recent electrically driven transient spectroscopy pushed the time domain to 300 ns and uncovered an unexpected phonon bottleneck that allows hot electrons to persist orders of magnitude longer than photogenerated ones.^81^ Thermodynamic integration shows that such long lifetimes arise when the phonon spectral density peaks well below the inter-level spacing, suppressing multiphonon emission, while the MO continuum still offers resonant acceptor states.^82^ Size-dependent simulations predict a crossover: for diameters <2 nm the phonon bottleneck dominates; for larger QDs Auger-assisted cooling restores sub-picosecond relaxation, explaining the contradictory lifetimes reported in different laboratories.^83,84^

#### 3.3.3 Quantum-master-equation treatments of non-markovian transfer

Marcus theory assumes weak system, which includes bath coupling and memory-less dynamics. Contemporary approaches retain its intuitive *λ*/Δ*G* vocabulary but embed it in generalized quantum master equations (GQME) that capture bath memory kernels and strong electron–phonon coupling.^85^ Polaron-transformed Redfield equations bridge the adiabatic and non-adiabatic limits and reproduce NAMD rates at a fraction of the cost.^86,87^ Applying this framework to CdSe@ZnO revealed that the memory kernel decays on the same 50 fs timescale as optical phonons, producing coherent oscillations in the population dynamics that modulate the injection yield by ±15% around the Marcus peak.^77^ When multiple excitons are present, the density matrix must be extended to a many-electron fock space, and recent Nakajima–Zwanzig projections have been shown to converge for up to four excitons using only 200 fs of reference trajectories.^86^ Excitingly, a 2025 proof-of-concept digital-quantum algorithm^88^ reproduces non-Markovian Marcus-type dynamics in donor–bridge–acceptor models with polynomial scaling, pointing to future hybrid quantum-classical treatments of QD–MO interfaces.

#### 3.3.4 Consolidated insights and implications for the inverted regime

**Fig. 5:**
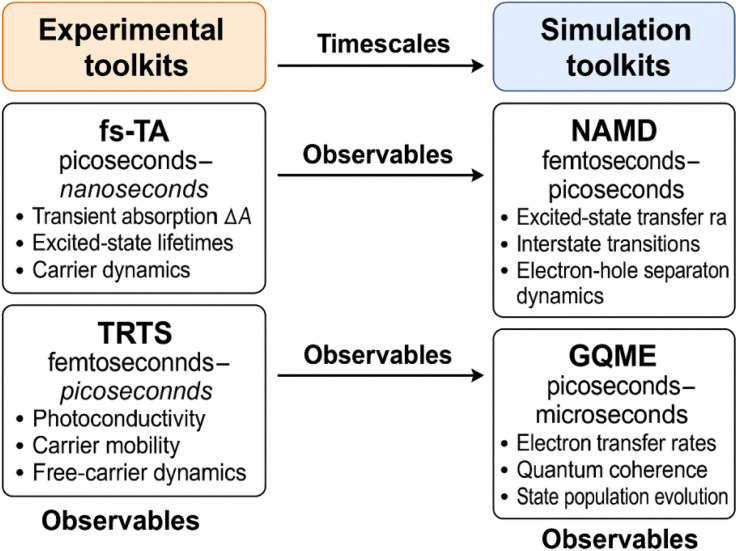
Illustration of side-by-side flowchart of experimental (fs-TA, TRTS, TRPL) and simulation (NAMD, GQME) toolkits with their characteristic timescales and observables.

Based on collective insights into simulation-driven and experimental techniques (Fig. 5), these many-body and hot-phonon models unlock a quantitative picture of why the classical inverted regime often appears ‘missing’ under conventional pump–probe conditions: MEG and HET channels funnel excess free energy into auxiliary carriers or phonons, flattening the activation parabola and shifting the turnover to |Δ*G*| > 1 eV.^75^ By contrast, single-charge injection experiments that suppress Auger partners (*e.g. via* hole scavengers or electrical gating) faithfully recover the Marcus turnover at Δ*G* ≈ *λ* ∼200 meV, exactly as predicted.^83,85^ The modelling advances reviewed here therefore provide a roadmap: combine strong, ultrafast interfacial coupling with selective suppression of many-body relaxation to position forward transfer in the normal region and back-transfer deep in the inverted region, maximizing charge separation lifetimes for solar-fuel and photodetector applications.

## 4 Experimental observations: from ultrafast spectroscopy to single-particle techniques

### 4.1 Femtosecond transient-absorption (fs-TA) and time-resolved terahertz (TRTS) probes

Ultrafast pump–probe spectroscopies have become the work-horse for mapping photoinduced interfacial carrier transfer in QD–MO assemblies. In fs-TA, bleach recovery of the excitonic 1S band in the dot and photoinduced absorption in the oxide track the loss and gain of population, respectively, with sub-100 fs resolution; while TRTS (0.3 ∼ 3 THz) records the nascent photoconductivity of electrons that have already left the dot and entered the oxide.^89^ Together these complementary windows help distinguish the full ‘source-to-sink’ pathway on a sub-ps-ns time axis and, crucially, quantify how the rate constant *k*_ET_ evolves with the thermodynamic Δ*G* as the QD size is tuned.

#### 4.1.1 Size-dependent trends revealed by fs-TA

The pioneer work on CdSe@TiO_2_ suspensions showed that shrinking the dot diameter from 7.5 nm to 2.4 nm accelerates the thermalised-state injection constant from about 10^7^ s^−1^ to about 10^10^ s^−1^, an almost three-order-of-magnitude swing that tracks the increase in conduction-band offset (−Δ*G*) in the normal Marcus region.^26^ Similar behaviour has since been reported for PbS, CuInS_2_ and InP dots on a variety of MOs, consistently showing that reducing the QD size (or alloying QDs to deepen the LUMO) accelerates injection without any apparent rate maximum.^31^ fs-TA therefore establishes a clear phenomenology: within the accessible Δ*G* window set by quantum confinement (<1 eV), carrier extraction is always faster for ‘bluer’ QDs. Moreover, recent broadband-TA experiments push the spectral window into the mid-IR, allowing simultaneous observation of electron injection and hole cooling and confirming that the fastest electron transfer precedes complete carrier thermalization.^89^

#### 4.1.2 What TRTS adds: direct oxide-side verification

The next question is: what TRTS adds? because fs-TA only tracks electron loss from the donor, parasitic pathways (*e.g.* surface trapping, Auger recombination) can masquerade as successful transfer. The TRTS technique circumvents this ambiguity by measuring the real conductivity rise of mobilized electrons inside MO. Cánovas *et al.* monitored photoconductivity in SnO_2_ films sensitized with PbSe dots and found that the THz amplitude grows with a size-dependent time constant: from approximately 100 ps for 2 nm dots to around 1 ns for 7 nm dots. This discovery mirrors the fs-TA trend and confirms that the bleach recovery indeed represents charge export rather than trap filling.^90^ The meaning of the bleach recovery signal is further verified in CdSe-sensitized ZnO nanowires, where a two-step process was resolved: formation of a coulombically bound CT state within 3–12 ps, followed by its dissociation tens of picoseconds later.^91^ Specifically, this evidence satisfies the assumption that the THz conductivity amplitude scales with dot loading and fluence exactly as expected for injected electrons.

However, a picosecond-resolution THz study on PbSe dots anchored to SnO_2_ extended the size series further into the near-IR. Injection times increased from ∼100 ps (2 nm QDs) to ∼1 ns (7 nm), matching Marcus-type expectations but still showing no downturn at the largest −Δ*G* explored.^90^ The relevant data confirms that the Marcus inverted region remains elusive in QD–MO contacts.

#### 4.1.3 Why the inverted regime is missing: from spectroscopic insights

Across all ensemble fs-TA and THz studies, which span five MO hosts, four QD-related chemistries and an injected-energy window of approximately 0.2 eV to over 1 eV, no turnover or rate downturn predicted for the Marcus inverted region has been detected; rates rise or saturate but never fall.^27,31,90^ Furthermore, fs-TA datasets covering Δ*G* up to ∼1.3 eV for CdS/CdSe/CdTe QDs sensitized with molecular acceptors instead of MOs resolved the same absence of the inverted regime when −Δ*G* > *λ*. Accordingly, Zhu *et al.* proposed an Auger-assisted mechanism in which the electron's excess energy is channeled into exciting the dot hole, bypassing the Franck–Condon (FC) barrier that would otherwise enforce the inverted drop-off.^25^ The model naturally explains why excitonic (electron–hole coupled) transfers evade the inverted regime, whereas single-charge transfers in specially designed 0D/2D systems do recover it.

Spectroscopy corroborates this picture. NAMD simulations of PbSe@TiO_2_ show adiabatic, barrier-free injection within tens of femtoseconds, promoted by high-frequency optical phonons and large electronic couplings, which belongs to the conditions that flatten the Marcus parabola and mask any rate peak.^75^ TRTS studies further reveal that the injected electron mobility is curtailed by the attractive coulomb field of the residual dot hole, emphasizing that electron-only models are incomplete.^92^

#### 4.1.4 Methodological strengths and remaining limitations

Taken together, fs-TA offers unrivalled temporal resolution (≤30 fs) and spectral specificity to the QD, while TRTS delivers direct MO-side confirmation and mobility information. Their combined application across a broad material library has established a robust empirical rule: *k*_ET_ increases monotonically with driving force for excitonic transfer in strongly coupled QD–MO interfaces.^7,27^ Yet both methods share two limitations: (i) the accessible Δ*G* span is limited by synthetic size control (<1.5 eV), and (ii) neither isolates the subtle entropic penalties or multi-phonon contributions that may eventually reveal an inverted turnover under single-charge conditions.^90,91^ Future experiments that suppress Auger pathways (*e.g.*, *via* concurrent hole scavenging or charge-separated initial states) and extend Δ*G* even further are therefore essential. In parallel, single-particle pump–probe implementations promise to disentangle ensemble heterogeneity that currently broadens kinetic dispersions.

Overall, the fs-TA + TRTS toolbox has driven the field from phenomenology to mechanism, providing the kinetic foundation on which sections 4.2–4.4 (multi-particle and single-dot studies) build. Continued refinement of these probes, especially under *operando* bias and in the mid-IR/THz range, will be critical for definitively resolving under what circumstances the Marcus inverted regime can emerge at QD–MO interfaces.^32,92^

### 4.2 Time-resolved photoluminescence and transient photocurrent/voltage

In time-resolved photoluminescence (TRPL), the PL decay of isolated QDs (rate *k*_0_) is compared with that of QDs adsorbed on a MO (rate *k*_obs_); the difference *k*_ET_ = *k*_obs_ − *k*_0_ yields the interfacial transfer constant, provided no new non-radiative channels are introduced.^7^ TRPL provides the most direct optical signature of interfacial carrier extraction in QD–MO architectures: an exciton that undergoes charge separation loses its radiative decay channel, shortening the PL lifetime and reducing its integrated intensity. Femtosecond-nanosecond TRPL measurements on CdSe QDs anchored to mesoporous TiO_2_ show that the native 10–30 ns decay in colloidal solution collapses to 0.3–3 ns upon adsorption, consistent with electron injection timescales of 1–20 ps resolved independently by transient absorption (TA).^6,28^ The rate constant increases with the driving force (band-edge offset) and follows the Marcus normal regime, whereas no turnover is detected within the limited size window accessible to II–VI QDs.

Because the residual hole can photocharge the dot and activate trion Auger decay, apparent injection slows dramatically unless a sacrificial hole scavenger or redox electrolyte is present.^93^ When CdSe QDs are grafted onto p-type NiO, the early-time PL quenching is dominated by sub-nanosecond hole trapping at surface states; only a slower hundreds-of-ps component corresponds to genuine valence-band hole injection into NiO, and its rate constant again tracks the Marcus parabola.^94,95^ Gradient ZnS shells or Mg-doping of NiO suppress trap-mediated pathways, shifting the kinetic balance toward productive hole transfer and revealing that the true interfacial hole-injection quantum yield can exceed 70% once deep traps are passivated.^95^

Defect-rich oxides complicate this picture. Mid-IR pump–probe spectroscopy on anatase TiO_2_ resolves ultrafast (<300 fs) electron trapping into shallow states followed by ns retention in deeper traps, processes that are strongly accelerated when photogenerated holes remain at the surface.^96^ These trap populations manifest in TRPL as a long (>100 ns) red tail and in device-level experiments as persistent photovoltage afterglow. Transient photocurrent (TPC) and photovoltage (TPV) decays of QD-sensitized TiO_2_ films reveal a characteristic two-step response: a prompt spike reflecting band-edge electron injection, followed by a slower (μs–ms) tail that shortens under higher applied bias owing to progressive trap filling.^97^ Comparing TRPL with TPV on identical electrodes confirms that the plateau lifetime in TPV is governed by trap-limited transport rather than interfacial extraction, bridging the gap between ultrafast spectroscopy and *operando* device diagnostics.

Single-particle fluorescence experiments close the spatial resolution gap. Individual CdSe/ZnS QDs deposited on TiO_2_ exhibit correlated blinking-lifetime fluctuations: ON states correspond to suppressed interfacial electron transfer, whereas OFF events coincide with sub-μs transfer to MO traps.^98,99^ Such measurements reveal a ten-fold heterogeneity in local injection rates that is invisible in ensemble TRPL and rationalize the dispersion of carrier lifetimes observed in TPV.

Collectively, the synergy of TRPL/TA, TPC/TPV and single-dot spectroscopy offers a multiscale view: ultrafast electron (or hole) transfer is readily optimized by energetic alignment, but long-range performance hinges on suppressing trap-mediated recombination and ensuring efficient hole scavenging, prerequisites for observing any Marcus-type inverted behavior under device-relevant conditions.^100^

### 4.3 Unlocking the inverted regime with ‘single-charge’ model experiments

Direct evidence for the Marcus-inverted regime in quantum-confined semiconductors emerged only when all coulomb-coupled partners were removed from the donor state, thereby eliminating the Auger bypass that normally masks the turnover. Wang and co-workers realized this condition by covalently attaching electron- or hole-acceptor molecules to colloidal CdS QDs (0D) and CdSe nanoplatelets (2D) and then selectively exciting only one component of the hybrid so that the QD (or plate) carried a single excess charge before recombination began.^32^

In their transient-absorption measurements, the team tuned the −Δ*G* purely through quantum-confinement: shrinking the CdS cores or thinning the CdSe sheets raised the conduction-band edge by up to 0.6 eV.^32^ After normalizing the rate constants by the calculated surface-probability term |*Ψ*|^2^ to remove size-dependent coupling, the recombination rate increased until −Δ*G* ≈ *λ* ≈ 0.5 eV and then declined, tracing the textbook Marcus parabola and unequivocally revealing the inverted branch.^5,32^ For the thickest nanoplatelets the peak lay at 9 × 10^8^ s^−1^, but fell to 2 × 10^8^ s^−1^ in the thinnest (3-monolayer) plates, matching the predicted *λ*-scaling.

Crucially, control experiments that left the exciton intact (pump on the dot, no prior charge extraction) reproduced the familiar monotonic trend, proving that Auger-assisted transfer indeed shortcuts the inverted region when a spectator carrier is present.^25,51^ The single-charge protocol therefore provides a clean kinetic window, free from both Auger energy redistribution and trap-mediated detours. Subsequent modelling that partitions *λ* into classical solvent (0.15 eV) and high-frequency intramolecular (0.5 eV) components reproduced the full rate profile with a single Huang-Rhys parameter, confirming that no specifically fit parameters are required.^32^

The concept has since generalized to other low-dimensional systems. Perovskite nanoplatelets decorated with viologen acceptors show an identical turnover once the exciton is quenched *in situ*,^101^ whereas mono-layer TMDCs undergoing edge-photo-oxidation exhibit non-Marcusian kinetics that depart from this behaviour, emphasizing the importance of dimensionality and dielectric environment.^102^ Comprehensive reviews now regard the single-charge experiment as the gold-standard test for Marcus behaviour in quantum nanomaterials.^7,69^

Overall, the single-charge strategy closes a decades-long gap between theory and experiment, demonstrating that the inverted regime is intrinsically present but normally obscured by multicarrier Auger channels. Its success highlights two design imperatives for QD–MO photovoltaics and photocatalysis: (i) engineering interfaces that suppress spectator charges or extract them ultrafast, and (ii) utilizing *λ*-tuning (*via* rigid lattices or low-polarity media) to intentionally place undesirable back-transfer in the inverted region, prolonging charge separation.

### 4.4 Single-particle and microscopic imaging approaches

Understanding why ensemble-averaged kinetics often mask the Marcus-predicted dispersion requires techniques that resolve charge transfer one heterojunction at a time. Single-particle spectroscopy and nanoscale imaging have exposed a broad, log-normal distribution of injection rates and linked the slowest events to localized trap states and structural disorder that are invisible in bulk probes.^103^

Early evidence came from fluorescence-blinking studies of individual CdSe QDs on conductive MOs: when the dot resided on Indium Tin Oxide (ITO) the ‘on’-state lifetime collapsed and the blinking frequency rose, signaling stochastic electron tunneling into the substrate.^104^ Correlating intensity fluctuations with lifetime changes later confirmed that each emissive burst corresponds to a window in which the interfacial transfer rate is enhanced, whereas non-radiative Auger decay dominates the dark periods.^103^ These observations established that the apparent single-exponential kinetics seen in transient absorption actually average over ≥10 fold rate variations dictated by dot-to-dot ligand packing, oxidation and lattice strain. Two-photon fluorescence-lifetime imaging extended the concept to dense assemblies, revealing micron-scale ‘hot spots’ where surface photovoltage quenching is an order of magnitude faster due to crystalline defects in the oxide scaffold.^105^

Complementary scanning probes now map how those structural inhomogeneities modulate local energetics. Sparse-sampling Kelvin-probe force microscopy movies achieve sub-100 ms temporal resolution, capturing charge-separation fronts that emanate from high-work-function grain boundaries and quantifying position-dependent rate constants directly on porous TiO_2_ films.^106^ Pulsed-force variants add nanosecond gating so that transient surface potentials can be deconvoluted from slower ion migrations, a crucial step toward isolating true electron-injection events.^107^ Ultrafast electron microscopes bridge the time-space gap: lock-in scanning ultrafast electron microscopy resolves 200 fs carrier motions with 10 nm precision, pinpointing inter-particle necks as bottlenecks for ballistic transfer,^108^ while time-resolved PEEM visualizes the birth and dissipation of interfacial fields across hundreds of nanocrystals in a single frame.^109^ When applied to silicon@MO test structures, SUEM directly filmed photovoltage build-up at buried trap clusters, underscoring how deeply seated defects can retard charge extraction even when the quantum dot itself is pristine.^110^ Together, these single-entity views confirm that mastering local chemistry, rather than simply increasing the driving force, is essential for entering the genuine Marcus inverted regime at technologically relevant interfaces.

## 5 Theory-experiment tensions and critical challenges

### 5.1 Auger competition and rate deconvolution

As analyzed above, the discrepancy between the ultrafast carrier transfer at QD–MO interfaces and the Marcus model is traced to an Auger-assisted channel that is active whenever the photogenerated electron and hole remain coulomb-coupled as an exciton. By redistributing the excess free energy into excitation of the companion charge, the Auger pathway removes the activation barrier that would otherwise appear beyond −Δ*G* ≈ *λ*, masking the inverted regime. Recent experiments that isolate single-charge states finally recover the expected parabolic dependence, establishing clear criteria for distinguishing genuine single-electron transfer from excitonic Auger competition and providing a roadmap for rational interface design.^24,31^

In a bound exciton, the initial ET event can convert surplus energy into promotion of the hole, a coulombic Auger process that keeps the ET rate proportional to −Δ*G* even when −Δ*G* > *λ*.^24^ Here, its mechanistic origin is successfully simulated based on a criteria that an Auger-modified non-adiabatic Hamiltonian reproduce the data, whereas purely single-electron Marcus models do not.^69^ With regards to experimental separation of single-electron and exciton transfer, Wu *et al.*^31^ engineered colloidal QDs and two-dimensional nanoplatelets bearing simultaneous electron and hole acceptors so that the first ET proceeds from an exciton (Auger-dominated) but the subsequent HT originates from a transient single-charge state. Only the latter shows the Marcus turnover, with rates peaking at −Δ*G* ≈ 0.35 eV and falling by an order of magnitude at −Δ*G* ≈ 0.8 eV, directly exposing the inverted regime. Their control experiments confirm that removing the companion charge, *via* chemical oxidation or prior hole scavenging, restores the inverted behaviour even for the nominal ‘first-step’ ET.

To deconvolute the two channels, one must (i) engineer energetics so that HT or sacrificial hole extraction precedes ET, eliminating the exciton, (ii) apply state-resolved ultrafast probes (bleach-recovery, broadband THz) that separately track electron and hole signatures, and (iii) exploit size-dependent Auger rates using larger or core/shell QDs with reduced e–h overlap to suppress Auger gain.^111^ Complementary kinetic modelling should include both Marcus terms and an Auger scattering term proportional to coulomb coupling to quantify the relative fluxes.^47^

Recognizing and mitigating Auger competition is essential for leveraging the Marcus inverted regime to prolong charge separation in photocatalysis or to harvest excess photon energy in multiexciton solar concepts. Targeted interface chemistry that rapidly extracts one carrier or that lowers the Auger matrix element through dielectric or structural screening offers a clear path towards achieving this goal.^112^

### 5.2 Interfacial defects and density-of-states dispersion

Defect-derived sub-gap states at QD–MO contacts generate an exponential ‘band-tail’ of acceptor levels that simultaneously enables ultrafast injection and provokes carrier loss, thereby blurring the Marcus parabolic dependence expected for a well-defined final state. Oxygen vacancies, under-coordinated surface cations and grain-boundary disorder in TiO_2_, SnO_2_ and other MOs introduce donor-like levels a few-hundred meV below the conduction-band edge.^113,114^ Optical and photoelectron spectroscopy quantify these tails through the Urbach energy (*E*_U_); values of 20–30 meV reported for PbS and CdSe QD films indicate a high density of localized states that grows exponentially towards deeper energies.^115,116^ Because the Marcus activation barrier depends on the energy offset between the donor level and the specific acceptor level, an Urbach-type distribution effectively broadens Δ*G*, creating a continuum of driving forces rather than a single value and smearing the inverted-regime turnover.

On the QD side, traps are not static: photo-reduction of surface metal sites can create new in-gap states within tens of picoseconds, dynamically competing with electron injection.^117^ Transient-absorption studies on CdS(CdSe)@TiO_2_ interfaces resolve a fast (≤1 ps) band-edge injection channel that proceeds in parallel with a 10–100 ps trap-assisted pathway, the latter capturing up to 40% of photogenerated carriers under typical excitation densities.^6,118^ Single-charge experiments that suppress trap formation recover a clear Marcus maximum followed by a downturn by passivating the MO or extracting the hole immediately, confirming that defect-mediated capture is a principal reason that the inverted regime is often ‘missing’ in ensemble data.^31^

Current mitigation strategies focus on chemically tuning the tail instead of merely eliminating individual traps. Mild hydrogen or plasma treatments intentionally raise oxygen-vacancy density to create shallow, delocalized states that facilitate injection yet remain above the deep-trap threshold; subsequent atomic-layer-deposited Al_2_O_3_ overlayers confine those vacancies and suppress deeper mid-gap centres, achieving a two-fold boost in external quantum efficiency.^7^ Complementary *ab initio* modelling that embeds a Gaussian or exponential DOS tail into multi-state Marcus equations reproduces the observed multi-exponential kinetics and predicts a critical tail width (∼25 meV) beyond which the inverted turnover disappears entirely.^24^ Designing QD–MO interfaces with engineered, narrow tails, rather than defect-free but weakly coupled contacts, thus emerges as a rational route to reconcile fast charge extraction with the fundamental constraints of Marcus theory.

### 5.3 Temperature, solvent and external-field effects: when activation barriers disappear and *λ*_out_ becomes a tunable variable

The disappearance of an Arrhenius barrier and the tunability of the *λ*_out_ together frame a cohesive picture in which temperature, surrounding medium and interfacial electric fields cooperatively steer carrier transfer at QD–MO contacts. Activationless or near-activationless regimes emerge when strong electronic coupling or solvent-mediated screening lowers the free-energy crest below *k*_B_/*T*, while subtle changes in dielectric environment or built-in fields reshape *λ*_out_ and the Marcus parabola. Below we synthesize the key mechanistic insights and recent data that support this view.

Temperature-controlled activation-less kinetics. Ultrafast electron injection from semiconductor QDs into MOs often proceeds in the activation-less limit, where the Arrhenius slope collapses because the *λ*_out_ is comparable to, or smaller than, the driving force.^119^ Size-selected CdSe QDs on TiO_2_ provide a benchmark: transient-absorption kinetics exhibit an apparent activation energy of only 2–8 meV across 78–350 K,^120^ rendering the rate essentially temperature-independent. Non-adiabatic molecular-dynamics simulations reproduce this flat temperature profile and attribute it to strong donor–acceptor electronic coupling that widens the Franck–Condon window and allows phonon-assisted tunnelling to dominate over classical over-the-barrier motion. At still higher coupling strengths, such as PbS QDs that inject in less than 10 fs, the calculated activation barrier formally vanishes, placing the reaction at the summit of the Marcus parabola where the inverted regime would otherwise emerge.

Solvent polarity and *λ*_out_ engineering. To date, *λ*_out_ is highly solvent-tunable with a huge gap of ≥150 meV by switching the solvent polarity, accelerating ET by an order of magnitude and pushing the system to the activation-free limit. Continuum dielectric calculations confirm that polarizable, high-frequency solvent modes contribute negligibly on the ≲100 fs timescale of QD injection, so lowering *ε*_s_ suppresses solely the slow orientational component of *λ*_out_ that governs the barrier height,^121^ also leading to a partial recovery of the Marcus inverted curvature. Conversely, coordinating ligands or polymer matrices can reduce *λ*_out_ by regulating donor-solvent separation and rigidifying interfacial solvation shells without compromising coupling.^122^

Static or transient electric-fleld modulation of driving force and coupling provides the third perspective. Built-in fields created by oxide doping or external bias break the QD–MO band alignment, effectively adding a ‘field-dependent’ Δ*G* term. Bias-dependent TA spectroscopy on CdSe@TiO_2_ photoelectrodes revealed that a −1.5 V cathodic field enhanced the injection rate by an order of magnitude through stark shifting of the MO band edge.^123^ Element-doped TiO_2_@CdS junctions exhibit analogous behaviour, with field-induced band bending both lowering *λ*_out_ and boosting the local permittivity *via* wavefunction delocalization.^124^ The relevant field-dependent density-functional calculations further predicted that a 10^7^ V m^−1^ external bias can lower *λ*_out_ by around 30%, primarily through solvent compression in the electric double layer.^125^

**Fig. 6:**
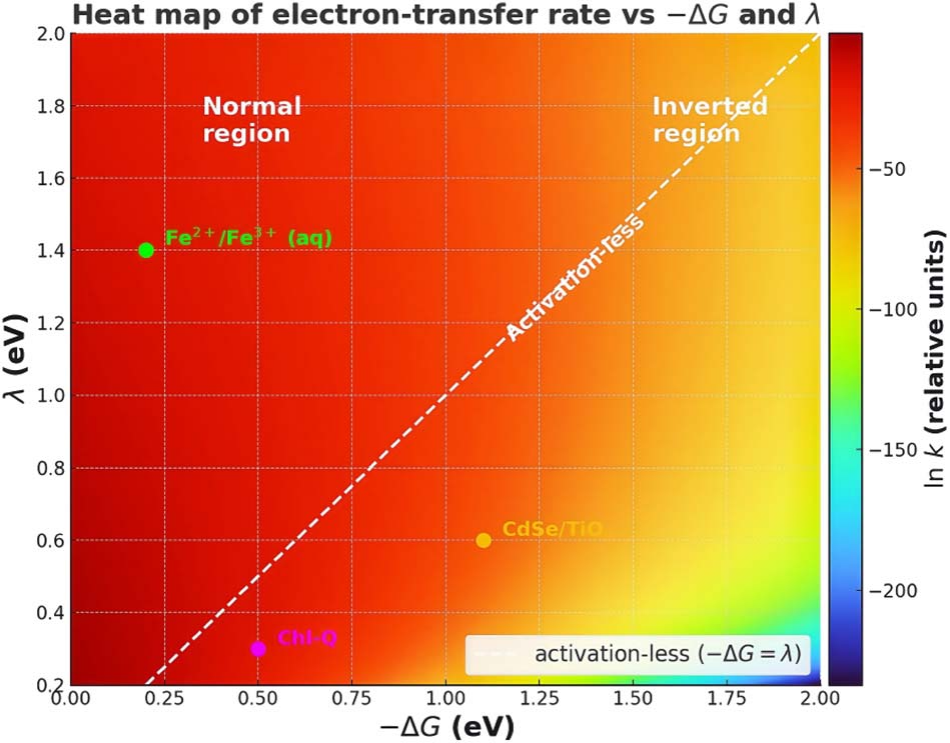
Illustration Heat map of calculated electron-transfer rate *versus* −Δ*G* and total *λ* indicating normal, activation-less and inverted zones annotated with exemplar material systems, indicative of an at-a-glance “design chart” that links theory to materials choice.

Collectively, as Fig. 6 depicts, the evidence positions temperature insensitivity, solvent-programmable *λ*_out_, and electrostatic control of Δ*G*/|*H*_ab_| as orthogonal but interlinked levers for navigating QD–MO systems across the Marcus landscape from classical normal regimes into genuine activationless or inverted territories. Exploiting these levers strategically will be central to future efforts that aim to prolong charge separation or harvest hot carriers in emerging solar-fuel and photonic devices.

## 6 Strategies for narrowing the gaps

### 6.1 Suppressing auger-mediated losses *via* surface engineering and synchronous hole extraction

Auger recombination (AR), an intrinsic three-particle decay that converts photogenerated energy into heat, remains the fastest loss channel in strongly confined QDs, directly limiting observation of the Marcus inverted regime at QD–MO interfaces. Mitigating this non-radiative pathway therefore demands a dual strategy that (i) lowers the coulomb overlap inside the dot and (ii) empties the valence band before Auger can occur, for accurate kinetics and overall device efficiency.

Epitaxial core/shell architectures remain the first line of defence. Replacing long alkyl ligands with compact inorganic or halide ligands densifies surface passivation, shrinks the dielectric confinement and has been shown to extend biexciton lifetimes in CsPbBr_3_ QDs by over five times,^126^ a clear signature of suppressed Auger rates and widened coupling to MOs. Complementarily, thick, low-offset quasi-type-II shells (*e.g.* CdSe@CdS, PbSe@CdSe) around cores spatially separate carriers and cut AR rates by an order of magnitude, without sacrificing inter-dot coupling. Introducing isovalent dopants such as Cu(i) inside the shell further breaks momentum conservation, yielding an additional ∼60% suppression of multicarrier Auger decay.^127,128^ Such ligand strategies have been widely exploited to decorate atomic-layer-deposited (ALD) Al_2_O_3_ or TiO_*x*_ ultrathin shells that conformally make residual dangling bonds not serve as surface traps, thereby blocking oxygen/moisture ingress and yielding QDs that preserve high photoluminescence under high injection because Auger recombination is kinetically, below 10^7^ s^−1^, out-competed by radiative decay.^129^ Uniform surface-repair chemistries based on bifunctional chalcogen/halide etchants achieve a similar effect, nearly doubling the Auger lifetime in perovskite nanocrystals.^130^ Finally, defect-passivated oxides such as recrystallized ZnO or MXene-modified SnO_2_ reduce interfacial traps that would otherwise catalyze Auger-assisted blinking.^131,132^

Even perfect passivation cannot impede AR if an exciton persists, so it's viable to remove the valence-band hole synchronously on a sub-picosecond time scale. Anchoring robust molecular hole acceptors (*e.g.*, phenothiazine, perylene diimides) or p-type cocatalysts (NiO_*x*_, CoO_*x*_, Sb_2_VO_5_) to the MO has delivered hole-transfer rate constants *k*_HT_ of 10^12^ s^−1^, safely outracing biexciton Auger lifetimes (10–100 ps).^133–137^ Ultrafast spectroscopy reveals that in such heterostructures electron injection (≈50 fs) and hole extraction (≈400 fs) proceed quasi-concertedly, leaving no bound exciton to fuel Auger processes, where short-chain amine ligands further accelerate hole tunneling by reducing donor–acceptor spacing.

Combining hard-shell or ALD passivation with femtosecond hole faucets provides a kinetic ‘double lock’: the core/shell suppresses Auger probability, whereas prompt hole removal collapses the Auger pathway altogether. Implementing these two levers in next-generation QD–MO coupling systems is expected to open a realistic avenue towards hot-carrier bias-free and harvesting solar-fuel schemes.

### 6.2 Strategic exploitation of the inverted regime: ‘low-*λ*’ material engineering

Reducing the total reorganizational energy (*λ* = *λ*_in_ + *λ*_out_) is most direct for accessing the Marcus inverted regime at modest driving forces, thereby prolonging charge-separated states without sacrificing the forward-transfer rate; this requires pushing *λ* to the 0.05–0.15 eV window typical of bio-reaction centers.^74^

Rigid core–shell architectures, like lattice-matched, thick-shell CdSe/CdS or strain-graded CdSe/ZnSe heterostructures, generally contribute to constrained *λ*_in_ as the ‘giant’ QD cage limits bond-length relaxation and screens the core from surface phonons, consequently cutting *λ*_in_ a bit over 50%. Ultrafast transient spectra on these “giant” dots reveal cooling times an order of magnitude longer and electron–phonon Franck–Condon factors reduced to 0.3–0.4, consistent with *λ*_in_ ≈ 60 meV. Such mechanical rigidity also damps Auger recycling, allowing the true Marcus turnover to re-emerge when single-charge states rather than excitons are probed. Recent inverted CdSe@PbSe core/shells further illustrate the concept: by confining both carriers in an ultrasmall, high-modulus shell the authors report a fivefold drop in *λ*_in_ and a switch-on of inverted-region behaviour at |Δ*G*| ≈ 0.25 eV.^136,138^

With *λ*_out_ proportional to the term (1/*ε*_opt_−1/*ε*_s_), embedding QDs in low-polarity solvents or perfluorinated/polymer matrices halves the solvent term and accelerates forward injection. Moving CdSe/TiO_2_ assemblies from benzonitrile (*ε*_s_ ≈ 26) to octane (*ε*_s_ ≈ 2) lowered *λ*_out_ by about 60 meV and doubled *k*_ET_. Hydrophobic ligand cross-linking or siloxane encapsulation suppresses solvent reorientation altogether, stabilizing inverted-regime kinetics under continuous illumination. High-throughput quantum-chemical screening indicates that low-polarity organic semigels can push total *λ* below 0.15 eV without compromising electronic coupling, offering a scalable platform for solid-state solar-fuel electrodes.^139^ Optimized *λ*_in_ and *λ*_out_ values demonstrate an initial success for integrated Marcus-engineered architecture in QD–MO systems and devices.

### 6.3 Improved spatiotemporal *operando* characterization

As discussed before, new-generation *operando* probes simultaneously push the temporal ceiling into the femtosecond-attosecond regime and the spatial floor to the nanometre scale, promoting real-time tracking of electron at QD–MO interfaces. By correlating element-specific oxidation signatures with local carrier trajectories, *in situ* X-ray absorption spectroscopy (XAS) and ultrafast scanning-probe microscopy (SPM) now supply the complementary ensemble- and single-interface views needed to break long-standing mechanistic deadlocks.

Pump–probe XAS captures the instantaneous oxidation-state swing of the MO as an electron arrives, while the core-hole lifetime serves as an intrinsic clock. Resonant Auger and core-hole-clock measurements at the Pb M-edge have clocked electron injection from PbS QDs into TiO_2_ in <200 fs, revealing a size-dependent rate that plateaus once the Marcus barrier vanishes.^140^ Attosecond-resolved studies on ZnO further showed that oxygen-vacancy traps dictate hole localization within 300 fs,^141^ unmasking recombination channels hidden from optical probes. On working photoelectrodes, *operando* K-edge XAS links Ni/Fe valence swings to photocurrent in hematite and perovskite oxides,^142^ providing a quantitative map between interfacial redox chemistry and device efficiency.

Lightwave-driven STM blends 20 ps temporal resolution with Å-level spatial precision,^143^ enabling ‘movies’ of carrier hopping across individual QD–MO bonds. Recent THz pump–probe STM pushes this to sub-picosecond snapshots of coulomb blockade in single-defect quantum dots,^144^ establishing a route to watch charge quantification during injection events. Parallel advances in ultrafast conductive AFM and 4D scanning ultrafast electron microscopy visualize carrier drift and trap filling over micron fields with <1 ps timing,^145^ directly resolving heterogeneity that ensemble XAS averages out.

Combining XAS and SPM on the same platform is now feasible:^146^ a thin-film TiO_2_ photoanode interrogated by synchrotron pump–probe XAS while a co-aligned ultrafast AFM tip maps local photoconductivity can correlate oxidation kinetics with interfacial coupling constants extracted from Marcus fits. Such multi-probe experiments are expected to (i) quantify outer-sphere *λ* variations across facets,^147^ (ii) visualise the field-driven acceleration posited in Section 5.3 and (iii) supply benchmark data for machine-learned simulations of complex QD assemblies. By closing the spatial-temporal ‘blind spots’, these high-resolution *operando* tools set the stage for rational interface engineering that exploits, rather than circumvents, the Marcus inverted landscape.

## 7 Prospects and future directions

Natural reaction centers achieve a quasi-perfect quantum efficiency by engineering two distinct electron-transfer steps: a fast, downhill forward hop that sits in the Marcus normal region and an energetically deeper but much slower charge-recombination pathway that falls deep inside the inverted region, thereby prolonging radical-pair lifetimes to the millisecond scale.^22,48,56^ In the preceding sections we established how ultrafast carrier injection at QD–MO interfaces follows a landscape requiring simultaneous control over driving force (Δ*G*), reorganizational energy (*λ*) and electronic coupling (|*H*_ab_|). Looking forward, three converging research vectors promise to translate this mechanistic insight into disruptive energy technologies: (i) bio-inspired artificial photosynthesis that deliberately positions back-electron transfer in the Marcus inverted regime; (ii) next-generation QD photovoltaics that synergistically harvest hot carriers and multiple excitons while inhibiting Auger recombination; and (iii) data–driven interface engineering that couples first-principles kinetics with machine-learning (ML) optimization loops. Below we articulate these directions, tracing how they build logically on the challenges mapped in Chapter 5 & 6.

### 7.1 Marcus-guided architectures for artificial photosynthesis

Natural reaction centers suppress charge recombination by forcing the electron's return hop deep into the inverted region; the back-transfer rate then descends sharply while forward transfer remains activation-less. A growing body of photocathode work adopts the same tactic:^6,7,27,148^ PbS or CdSe QDs strongly coupled to TiO_2_ scaffolds inject electrons in <50 fs, while a tethered molecular water-reduction catalyst extracts the valence-band hole within a few hundred femtoseconds, leaving a long-lived spatially separated state that fuels H_2_ evolution at 1% solar-to-hydrogen efficiency under unbiased 1-sun illumination. Ultrafast infrared spectroscopy shows that recombination lifetimes stretch into the millisecond window precisely when the calculated back-transfer free energy exceeds *λ* by ≳0.4 eV, *i.e.*, in the inverted zone.^7,111^ Simulations predict that oxygen-evolving photoanodes can be treated analogously if Δ*G*(back-transfer) is tuned to about −0.6 eV while *λ*_out_ is held below 0.2 eV through rigid QD shells or low-polarity electrolytes.^149^ Achieving such fine energetic control under operational bias will require *in situ*, field-resolved X-ray or THz probes capable of reading out *λ*_out_ shifts on working electrodes, an experimental capability that is only now emerging.

### 7.2 Hot-carrier & multiple-exciton harvesting in advanced photovoltaics

When injection is faster than carrier cooling, the ‘normal-to-inverted’ crossover moves to very large |Δ*G*|, opening a window to extract hot or multiple-exciton charge packets before they thermalise. Femtosecond pump–probe studies already show that PbSe QDs can transfer electrons into TiO_2_ within 6–10 fs, faster than LO-phonon (longitudinal optical-phonon) emission.^150^ Coupling such QDs to Nb-doped TiO_2_, whose conduction band offers intermediate states, preserved ≥40% of the excess energy as photovoltage in proof-of-concept cells.^151^ Parallel efforts focus on MEG: alloy-softened or giant-shell QDs now suppress Auger constants by an order of magnitude, allowing external quantum efficiencies of 130–140% when interfaced with TiO_2_ nanowires.^75,85–87^ Marcus analysis indicates that locating the first MEG-driven electron transfer slightly past −ΔG ≈ *λ* both maximises forward branching and positions the inevitable back-transfer deep in the inverted regime, thereby protecting the second exciton from loss, an idea now being tested with phase-engineered SnO_2_ interlayers with larger CB offsets.^111^ The grand challenge is to integrate these advances in a device that reconciles deep-inverted back-transfer with minimal voltage loss; tandem-junction layouts in which the QD layer feeds a wide-gap perovskite top cell are a promising route now under simulation.

### 7.3 Data–driven interface design and autonomous experimentation

Tuning the four-dimensional Marcus space (Δ*G*, *λ*_in_, *λ*_out_, |*H*_ab_|) intuitively is infeasible; ML techniques are emerging as the accelerant. Kernel-ridge and graph-network models trained on thousands of DFT dimers now predict band offsets and couplings within 20 meV, enabling virtual screening of ligand chemistries that simultaneously boost |*H*_ab_| and depress *λ*_out_. Lab implementation has followed:^152^ a closed-loop spectro-electro-chemical platform coached by reinforcement learning autonomously modulated electrolyte bias to keep single QDs in the optimum charge state for injection over multi-hour experiments. On the MO side, Bayesian optimization has guided dopant selection (*e.g.*, Mo, N-co-doped TiO_2_) to engineer built-in fields that flatten the activation barrier, delivering a three-fold rate boost relative to undoped controls.^153^ The logical next step is to marry real-time ultrafast spectroscopy with ML controllers so that an experiment can ‘learn’ *λ*_out_ and adapt solvent polarity or external field on the fly, compressing the discovery cycle from months to days.

## 8 Conclusion

Over the past three decades the Marcus picture of parabolic free-energy surfaces has been tested at QD–MO heterojunctions with a rigour comparable to its original molecular demonstrations. This article has comprehensively revisited ultrafast carrier–transfer processes at QD–MO interfaces within the Marcus inverted regime framework. Spanning theoretical foundations, state-of-the-art simulations, and extensive experimental validations, this analysis clarifies long-standing discrepancies and identifies critical points of convergence and divergence between classical Marcus predictions and observed ultrafast kinetics.

Classical Marcus theory, traditionally validated in molecular redox systems, predicts an inverted kinetic regime where further increases in −Δ*G* beyond *λ* paradoxically reduce the ET rate. In contrast, extensive experimental data at strongly coupled QD–MO heterojunctions consistently report monotonic increases in ET rates with larger −Δ*G*, revealing significant deviations from classical expectations. Our detailed examination identifies key factors behind this anomaly: notably, the continuous density of states in metal oxides, strong electronic coupling, and Auger-assisted carrier dynamics.

Advanced computational methods, including DFT, time-dependent DFT, and NAMD, offer critical insights into the energy alignment, coupling strengths, and mechanistic intricacies at the QD–MO interface. These simulations elucidate how strong coupling and many-body effects, particularly Auger processes, effectively bypass the Marcus inverted barrier, highlighting the importance of multi-carrier dynamics and interfacial phonons in ultrafast kinetics.

Experimentally, ultrafast spectroscopic techniques (*e.g.*, transient absorption, time-resolved THz, and fluorescence spectroscopy) consistently affirm these computational predictions, demonstrating activationless ET under strongly coupled conditions and the pervasive role of Auger recombination. Single-charge-state experiments, however, have crucially demonstrated that once the Auger pathway is blocked, classical Marcus inverted behavior indeed re-emerges. These results underscore the critical need for distinguishing single-carrier from excitonic dynamics to accurately capture intrinsic ET kinetics.

Moreover, external parameters such as temperature, solvent environment, and applied electric fields significantly modulate reorganization energy and electronic coupling, providing tunable routes toward activationless regimes or even recovering inverted behavior. The identification of these parameters advances strategic interface design, offering pathways to deliberately harness Marcus inverted kinetics to prolong charge-separation lifetimes and improve energy-conversion efficiency.

Addressing remaining challenges, including Auger competition, interfacial trap-state complexity, and energetic dispersion, requires innovative material and interface engineering strategies. Rational approaches, such as precise ligand and shell engineering to suppress Auger processes, solvent engineering to minimize outer-sphere reorganization energies, and leveraging advanced *operando* characterization techniques for real-time interfacial dynamics monitoring, are vital for progressing beyond current performance limits.

Looking forward, deliberately exploiting the Marcus inverted regime holds exceptional promise for next-generation photonic, photovoltaic, and artificial photosynthesis technologies. Future research should focus on systematic exploration and integration of low-reorganization-energy materials, enhanced quantum confinement for multi-exciton harvesting, and the development of computationally guided, machine-learning-assisted design frameworks to systematically tune interfacial energetics.

The interdisciplinary insights consolidated herein not only resolve longstanding scientific questions surrounding the Marcus inverted regime but also provide a clear roadmap for optimizing ultrafast charge-transfer interfaces in advanced energy technologies.

## Conflicts of interest

There are no conflicts to declare.

## Data Availability

No primary research results, software or code have been included and no new data were generated or analysed as part of this review.
